# Adaption and validation of the Perceived Control of Internal States Scale (PCOISS) in Chinese adults: a cross-sectional study

**DOI:** 10.1186/s40359-022-01004-2

**Published:** 2022-12-05

**Authors:** Yantao Chen, Zhuxi Yao, Naiyi Wang, Jianhui Wu, Yuanyuan Xin

**Affiliations:** 1 School of Psychology, Shenzhen University, Shenzhen, China; 2 Lab for Educational Neuroscience, Center for Educational Science and Technology, Faculty of Education, Beijing Normal University, Beijing, China; 3 Institute of Educational Psychology and School Counseling, Faculty of Education, Beijing Normal University, Beijing, China; 4 Center for Educational Science and Technology, Institute of Advanced Studies in Humanities and Social Sciences, Beijing Normal University, Zhuhai, China

**Keywords:** Perceived Control of Internal States Scale, Cross-sectional study, Psychometric validation

## Abstract

**Background:**

Perceived control of internal states is important for disease prevention, stress buffering and life adaptability. However, there is no psychometric scale to measure control beliefs over internal states in China. This study aimed to adapt and validate the Perceived Control of Internal States Scale (PCOISS) in a large sample of Chinese adults.

**Methods:**

Data was collected through a big project, in which a cross-sectional online survey was conducted nationwide in China using a powerful Chinese online survey platform named WenJuanXing (https://www.wjx.cn/). We translated the PCOISS into Chinese (C-PCOISS) with the forward–backward translation procedure. For the first time of the survey, a sample of 2709 participants (Sample 1) was valid for final analysis. Sample 1 was split into two datasets for principal component analysis (PCA) (n_A_ = 1355) and confirmatory factor analyses (CFA) (n_B_ = 1354) to determine potential factor structure. The scale’s validity (i.e., discriminant validity, convergent validity, criterion validity) and internal consistency reliability were evaluated. Among the 1354 respondents (n_B_), 761 (n_C_ = 761) participated in the follow-up second wave of the survey to assess a cross-sectional test–retest reliability.

**Results:**

The C-PCOISS retained 14 items. PCA yielded a three-factor model which was supported with the best fit indices in CFA. The C-PCOISS had satisfactory internal consistency with Cronbach’s alpha coefficients of 0.86, 0.78 and 0.72 for three subscales, respectively. The scale also showed adequate test–retest reliability (Pearson correlations coefficient of 0.64, 0.62 and 0.54 with *p* < 0.001 for three subscales, respectively). Three factors of the C-PCOISS were positively associated with positive affect, and negatively associated with negative affect, depression, compulsion-anxiety and perceived stress.

**Conclusions:**

The C-PCOISS is reliable and valid for measuring control beliefs over internal states in Chinese adults.

**Supplementary Information:**

The online version contains supplementary material available at 10.1186/s40359-022-01004-2.

## Background

Perceived control is generally considered as the degree to which one believes that situations or events can be influenced or controlled by their actions [1–4]. As a central construct in psychology [5], perceived control is more stable and persistent than actual control [6], which is particularly important for physical health and mental well-being [5, 7–10]. Perceived control can be divided into control beliefs over external events (e.g., perceived control over stressful events and perceived behavioral control) [5, 11], and control beliefs over internal states (e.g., perceived control of motivations, emotions, thoughts and physical well-being) [12].

Over the past two decades, control beliefs over internal states have been found important in many different fields. Firstly, it has a significant impact on the disease formation and development. Studies found that higher perceived control of emotions rather than disease course predicted lower depression in patients with cancer or HIV infection [13, 14], whereas people with lower perceived emotion control were more vulnerable to generalized anxiety disorder [15, 16]. Secondly, it has been demonstrated as a protective factor against stress. There is evidence that higher perceived control of internal states can facilitate the elderly with better psychological adjustment under mild life stress [17], buffer the adverse effect of academic stress on attention process [18], and alleviate psychological distress during quarantine [19]. Further, higher control beliefs over internal states were also found associated with better life resilience, such as greater life satisfaction [20] and higher competence [21]. Because of all these significant associations, perceived control of internal states has been taken as a great tool to assess the effectiveness of some clinical interventions or treatments [22, 23]. Due to its potential importance, we wish to introduce perceived control of internal states into China by adapting and validating a selected instrument.

There are varying terms with similar implications to perceived control of internal states, such as Locus of Control [24] and Self-efficacy [25], which also generated some validated scales including the Internal–External Locus of Control Scale (I-E Scale) [24] and the Generalized Self-Efficacy Scale (GSES) [26]. However, subtle differences between these concepts and perceived control of internal states should be noted. In the theory of Locus of Control, internal locus of control refers to the degree individuals believe their behavior is guided by personal efforts rather than other external circumstances like fate and luck [24], which mainly focus on the perceived causes of events and outcomes in external world. Self-efficacy refers to individuals’ beliefs in their capacity to produce specific performance attainments, in which only perceived competence for external events is specified [25]. Differently, perceived control of internal state focus on participants perceptions of their ability to influence their internal states and moderate the impact of aversive events on their emotions, thoughts, and physical well-being rather than an external world [13, 27]. The Perceived Control of Internal States Scale (PCOISS) was developed by Pallant [27] to measure personal perception of control over psychological states such as thoughts and emotions, which emphasizing the beliefs that one can quickly regulate emotions, get rid of bad thoughts, and moderate physical reactions.

Items in the PCOISS were initially developed through interviews with adults of varying backgrounds and finally concluded from three domains, i.e., emotion, thoughts and physical reactions [27]. Through two studies with 250 and 479 participants respectively, the reliability and validity of the PCOISS were supported [27]. Specifically, three factors and two factors were obtained respectively in their first study and second study, but the author finally combined the three or two factors into one as the factors were closely related and recommended the one-factor structure [27]. So far, the PCOISS has been translated into Turkish version [28] in which three-factor structure (i.e., ‘having the techniques for control of internal states’, ‘sense of efficacy of controlling internal states’ and ‘sense of lack of efficacies’) was validated. However, there is no Chinese version of the PCOISS, which impedes studies on the domain of perceived control of internal states in China and making international comparisons. Therefore, it is necessary to adapt and verify this scale to the Chinese population.

In this study, we aim to examine the psychometric properties of PCOISS among Chinese adults. We considered evidence for the reliability (i.e., internal consistency and test–retest reliability) and validity (i.e., construct validity, convergent validity, discriminant validity, and criterion validity). Similar to Pallant’s research [27], depression, anxiety, positive and negative affect, and perceived stress were used as criteria.

## Methods

### Study design

The data was collected through a big project, in which a cross-sectional online survey was conducted nationwide during the COVID-19 pandemic in China from January 31 to February 9 and from March 15 to March 18 in 2020 by WenJuanXing (https://www.wjx.cn/), which is a powerful Chinese online survey platform [29]. Self-reported questionnaires were distributed in the survey, including the Perceived Control of Internal States Scale (PCOISS) [27], the Perceived Stress Scale 10-item version (PSS10) [30, 31], the Psychological Questionnaire for Emergent Events of Public Health (PQEEPH) [32] and the Positive and Negative Affect Schedule (PANAS) [33]. Data from the same project has been also used in other studies [19, 34–36]. This study was approved by the Ethics Committee of Peking University.

### Participants

**Fig. 1:**
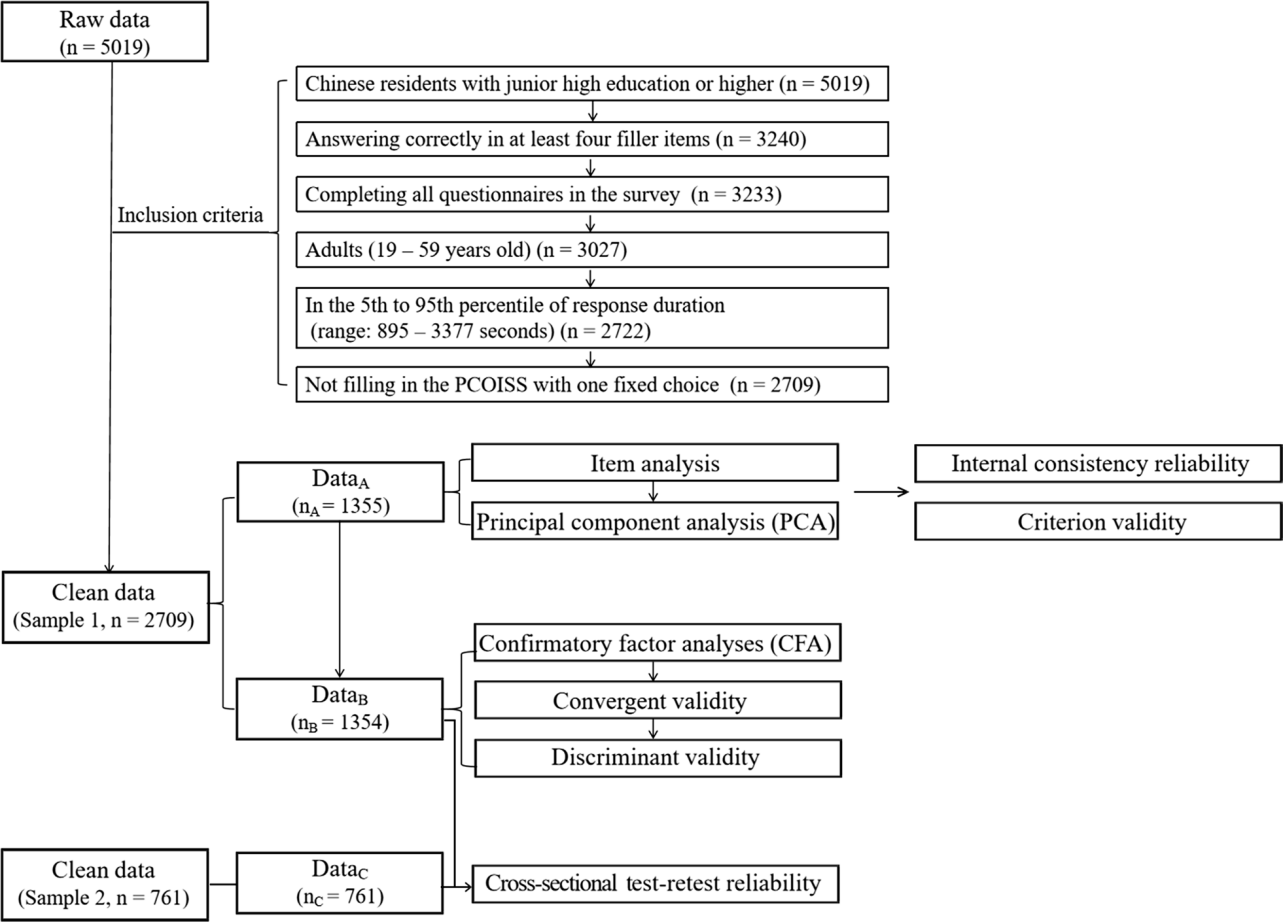
Flow chart of data processing

Data from 5019 participants was collected for the first time of the survey, of which 2709 (Sample 1; age = 32.10 ± 8.88 years; 1451 females) remained valid for subsequent analysis with following inclusion criteria (Fig. 1): (a) Chinese residents with junior high school education or higher (n = 5019); (b) answering correctly in at least four in six filler items (e.g., “I usually feel that winter is hotter than summer”) (n = 3240); (c) completing all questionnaires in the survey (n = 3233); (d) adults (19–59 years old) (n = 3027); (e) in the 5th to 95th percentile of response duration (range: 895–3377 s, mean = 1490.55 s, SD = 547.20 s, n = 2722); (f) not filling in the PCOISS with one fixed choice (e.g., selecting ‘3’ in the whole scale) (n = 2709).

Data of 761 primary participants (Sample 2; 443 females; age = 31.04 ± 8.60 years) from the follow-up second time of the survey was used to determine the test–retest reliability for the PCIOSS. Participants who completed all questionnaires received money compensate for each wave of the survey separately. All participants provided written informed consent.

## Materials

### Perceived Control of Internal States Scale (PCOISS)

The 18-item PCOISS measures the degree to which individuals feel they have control of their internal states [27]. Participants rated on a 5-point Likert scale ranging from 1 (strongly disagree) to 5 (strongly agree) for items such as “my feelings are usually fairly stable”. Higher scores indicate higher levels of perceived control of internal states.

### Translation of PCOISS and cross-culture adaption

Permission to adapt the PCOISS was obtained from the original author (Dr. Pallant). Following the guidelines for cross-cultural adaptation [37], the processes of translation from English to Chinese were as follows. Firstly, forward translations were made by two PhD students (native Chinese speakers) majored in psychology independently. Secondly, discrepancies between the two forward translation versions were discussed and identified to get a reconciled forward translation version. Thirdly, one bilingual (in English and Chinese) expert with good knowledge of psychology, blinded to the original version back-translated the reconciled forward translation. Finally, the backward translation was compared with the source version. Discrepancies were discussed and above steps were iterated to develop a final translated Chinese version (see Additional file 1: Table S1). Considering that the project investigated changes in mental health during the COVID-19 pandemic, we made some adjustments to fit with that context. For example, item 1 was revised to the sentence “I don’t have much control over my recent emotional reactions”, while item 4, 8, 9 and 10 were deleted the word “usually”.

### Perceived Stress Scale 10-item version (PSS10)

The 10-item PSS10 assesses perceived stress for the past one month [30, 31]. An example item is “have you felt nervous and stressed for the past one month?” Items were rated from 1 (not at all) to 5 (very much). The PSS10 demonstrated good internal consistency (α = 0.805) in the current sample.

### Psychological Questionnaire for Emergent Events of Public Health (PQEEPH)

The 27-item PQEEPH measures mental health status, which is adapted from the SARS Psychological Behavior Questionnaire (SARS-PBQ) [32] and includes five dimensions of depression, fear, compulsion-anxiety, neurasthenia and hypochondria. Considering that some items of SARS-PBQ were designed specifically for SARS, we made appropriate adjustments in the context of the current COVID-19 pandemic. For example, in the item “thinking of something to do with emergent events of public health, I was in no mood for anything else”, “emergent events of public health” was replaced with “the COVID-19”. Each item is rated from 0 (never) to 3 (severe). Consistent with Pallant [27], two dimensions of depression (e.g., “Less energy than before”) and compulsion-anxiety (e.g., “I felt my heart beat faster, sweated, and blushed”) were selected as criteria of PCOISS in further analysis. The Cronbach’s alphas for depression and compulsion-anxiety were 0.805 and 0.716.

### Positive and Negative Affect Schedule (PANAS)

The 10-item PANAS measures positive (e.g., “determined”) and negative (e.g., “upset”) affect for the past one week [33]. There are five items in each dimension. Items were rated from 1 (not at all) to 5 (very much). The Cronbach’s alpha for positive and negative affect were 0.767 and 0.779 separately in the current sample.

### Statistical analysis

Data analyses were performed using IBM SPSS 25.0 and Amos 24.0 software (IBM Corp., Armonk, NY, USA). Figure 1 shows the data analysis process. Sample1 (n = 2709) was split into two datasets, including Data_A_ (n_A_ = 1355, subject ID = 1355-2709) for PCA and Data_B_ (n_B_ = 1354, subject ID = 1-1354) for confirmatory factor analyses (CFA).

We conducted principal component analysis (PCA) with varimax rotation to determine the factor structure of PCOISS. Prior to PCA, we did the item analysis to make sure that every item is appropriate for factor analysis. Then we conducted Kaiser-Meyer-Olkin (KMO) test and Bartlett’s test of sphericity to warrant the data is suitable for factor analysis. Kaiser’s criterion (eigenvalues > 1.0) [38, 39], and Cattell’s scree test [40] were used to determine the number of factors.

After PCA, the constructs were verified by CFA with the maximum likelihood method. Criteria are as following: (a) goodness-of-fit index (GFI), comparative fit index (CFI) and Tucker-Lewis index (TLI) above 0.90 (acceptable 0.85); (b) standardized root mean square residual (SRMR) and (c) root mean square error of approximation (RMSEA) below 0.05 (acceptable 0.08) [41]. To avoid the problem that plausible models might be rejected [42], we compared two plausible models against the baseline model to examine the discriminant validity. First, the one-factor model was included as the author recommend one underlying factor in the previous work [27]. Second, a random intercept item factor analysis was used to control the impact of wording effect from those reversed scoring items [43]. The best model would be established by multiple combinations of fitting measures [44] including chi-square difference tests and information criteria computed as Akaike’s information criterion (AIC) [45], Bayesian information criterion (BIC) [46] and consistent AIC (CAIC) [47]. After the best model was selected, we calculated standardized factor loading and composite reliability to test convergent validity.

Internal consistency reliability was assessed with Cronbach’s alpha value. A cross-sectional test–retest reliability was also evaluated by Pearson's correlation coefficients with Sample 2 (n_C_ = 761) from the second survey. We also calculated Pearson correlations between three subscale scores of the Chinese version of the PCOISS (C-PCOISS) and criteria of interest to assess the criterion validity.

## Results

### Demographic characteristics of participants

The demographic characteristics of the total sample, Data_A_ and Data_B_ are presented in Table 1. Participants were on average 32.1 years (SD = 8.88). The majority of the participants were females (53.6%) and had education levels of college or higher (82.3%). An average response duration of the survey across participants was 24.84 ± 9.12 min.

**Table 1:** Demographic Characteristics of the participants

Variables	Total sample (n = 2709)	Data_A_ (n_A_ = 1355)	Data_B_ (n_B_ = 1354)
	Mean ± SD or n (%)	Mean ± SD or n (%)	Mean ± SD or n (%)
Age (years)	32.10 ± 8.88	33.19 ± 9.15	31.00 ± 8.46
Gender			
Male	1258 (46.4%)	664 (49.0%)	594 (43.9%)
Female	1451 (53.6%)	691 (51.0%)	760 (56.1%)
Education level			
Junior high school	143 (5.3%)	115 (8.5%)	28 (2.1%)
High school	336 (12.4%)	242 (17.9%)	94 (6.9%)
Junior college	525 (19.4%)	301 (22.2%)	224 (16.5%)
Bachelor degree	1466 (54.1%)	577 (42.6%)	889 (65.7%)
Master degree or above	239 (8.8%)	120 (8.9%)	119 (8.8%)

### Item analysis

All CRs by t-test between the upper and lower 27% of the group significantly exceeded 3 [48] and corrected item-total correlations were above the recommended level of 0.33 (see Additional file 1: Table S2) [49], suggesting that all items has adequate discrimination.

### Principal component analysis

**Fig. 2:**
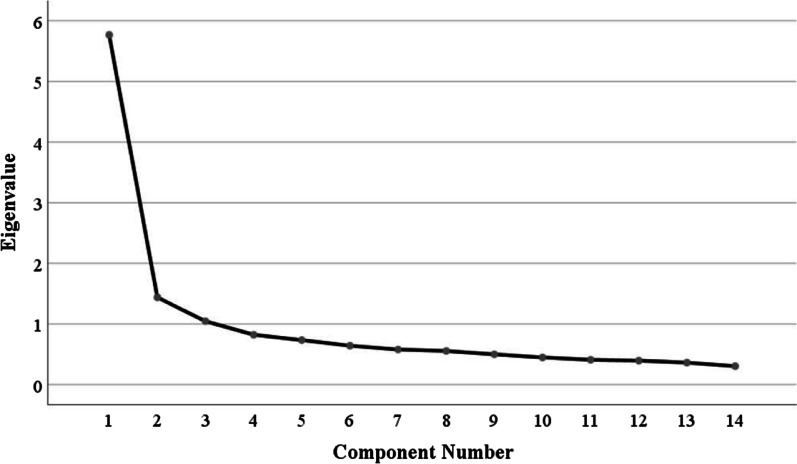
Scree plot for exploratory factor analysis of the C-PCOISS. The C-PCOISS, the Chinese version of the Perceived Control of Internal States Scale

KMO value was 0.933 (> 0.6) [50] and Bartlett’s test of sphericity was significant (*p* < 0.001) [51], suggesting the applicability of PCA. PCA was conducted iteratively until cross-loadings of the remaining items were less than 0.4. Item 2, 8, 1 and 6 were removed in turn due to larger cross-loadings values (> 0.4) [51], leaving 14 items in the end. As shown in Table 2, all 14 items had sufficient communalities (0.43–0.72, > 0.2) [48] and factor loadings (0.56–0.68, > 0.4) [51]. Three factors (explaining 58.93% of the total variance) with eigenvalues greater than 1.0 were extracted (see Fig. 2). Specifically, the first factor (item 12, 13, 14, 17, 18) accounted for 24.26% of the total variance, the second factor (item 3, 4, 5, 9, 10) accounted for 18.87% and the third factor (item 7, 11, 15, 16) accounted for 15.81%. These three factors were consistent with those of the previous Turkish version of the PCOISS [28]. Accordingly, we named them as ‘having the techniques for control of internal states’, ‘sense of efficacy of controlling internal states’ and ‘sense of lack of efficacies’. The final 14-item C-PCOISS is shown in Additional file 1: Table S1.

**Table 2:** Rotated component matrix for PCA of the 14-item PCOISS (n_A_ = 1355)

Item number	Communalities	Factor loadings		
		Factor 1: having the techniques for control of internal states	Factor 2: sense of efficacy of controlling internal states	Factor 3: sense of lack of efficacies
12	0.62	0.75	0.22	0.12
13	0.60	0.66	0.38	0.17
14	0.72	0.81	0.24	0.10
17	0.72	0.82	0.19	0.12
18	0.53	0.64	0.31	0.18
3	0.58	0.10	0.74	0.15
4	0.62	0.24	0.73	0.14
5	0.59	0.34	0.67	0.19
9	0.43	0.32	0.56	0.14
10	0.51	0.38	0.57	0.20
7	0.46	0.01	0.15	0.66
11	0.57	− 0.01	0.20	0.73
15	0.65	0.35	0.12	0.71
16	0.65	0.36	0.13	0.71

### Confirmatory factor analysis

**Fig. 3:**
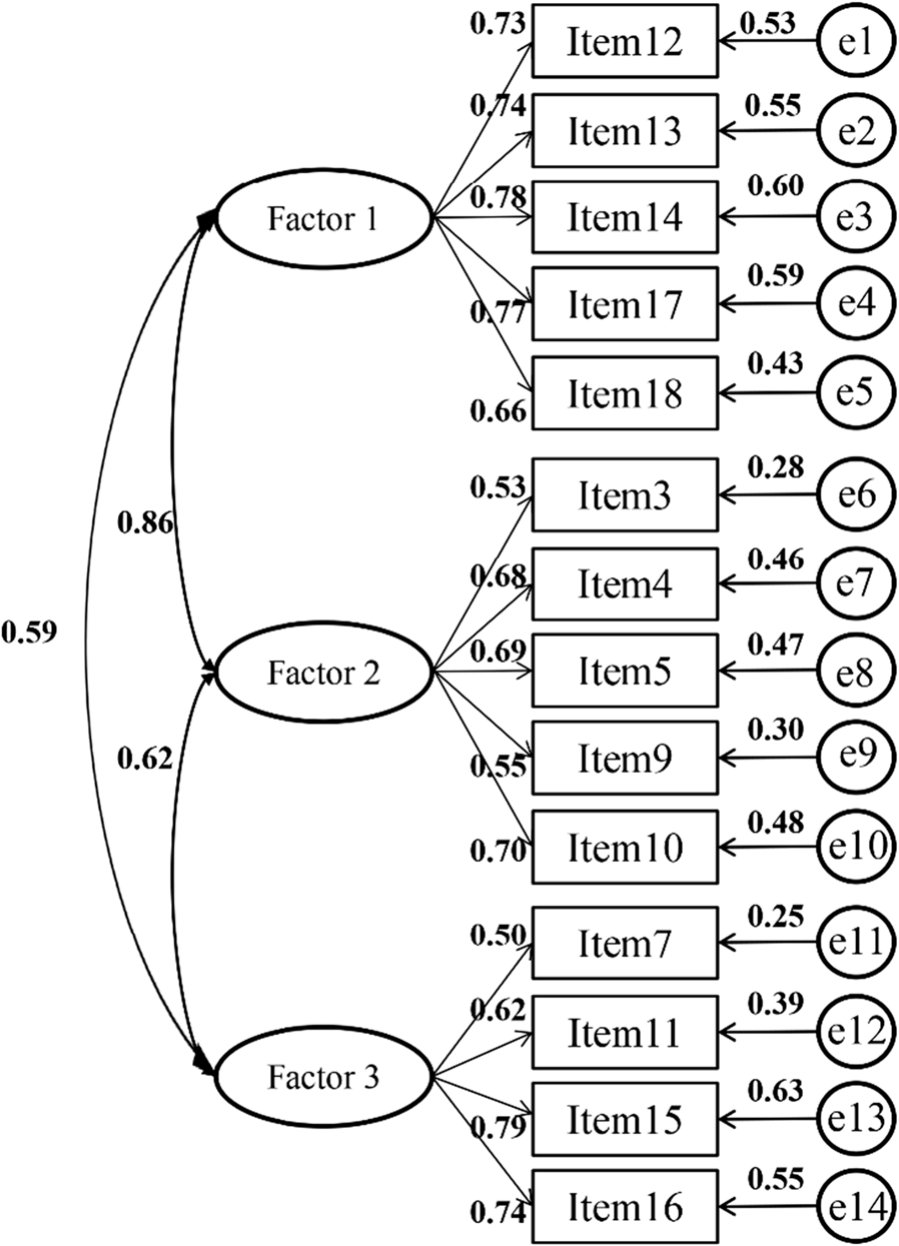
Standardized three-factor structural model of the C-PCOISS. The C-PCOISS, the Chinese version of the Perceived Control of Internal States Scale

Model fit for different models are presented in Table 3. Compared with Model 2, Model 1, the three-factor baseline model, had the best fit to the data (GFI = 0.96, TLI = 0.96, CFI = 0.95, RMSEA = 0.06, SRMR = 0.04; Chi-square difference test: *p* < 0.001; AIC, BIC and CAIC were minimum) [41]. Model 1 also fitted better than the random intercept factor model (Model 3), indicating the C-PCOISS is not affected by wording effect (see Additional file 1: Table S3). As shown in Fig. 3, the convergent validity for 14-item PCOISS was appropriate in standardized factor loadings (0.50–0.79, > 0.5) and composite reliabilities (0.77–0.86, > 0.7) [51].

**Table 3:** Fit indices and model comparisons (n_B_ = 1354)

Model	χ^2^	df	GFI	TLI	CFI	RMSEA	SRMR	AIC	BIC	CAIC	△χ^2^	△df
Three-factor model (Model 1)	406.01	74	0.96	0.94	0.95	0.06	0.04	468.01	629.54	660.54		
One-factor model (Model 2)	1245.73	77	0.86	0.81	0.84	0.11	0.07	1301.73	1447.64	1475.64	839.73***	3
Random intercept factor model (Model 3)	620.01	76	0.93	0.91	0.92	0.07	0.04	678.01	829.12	858.12	214.01***	2

Significance is indicated by (^***^) for *p* < 0.001. Model 1: Factor 1, 2 and 3 constituted the first-order oblique model. Model 2: Factor1, 2 and 3 were combined into one factor. Model 3: Each item was affected by a trait variable, a method effect variable and an error. Compared with Maydeu-Olivares and Coffman’s original model [43], the difference in this study was that reverse scoring items were used, for which the loadings were fixed to − 1 in the method effect variance

### Criterion validity

As shown in Table 4, three factors of the C-PCOISS were positively correlated with positive affect (r = 0.37, 0.33 and 0.16, *ps* < 0.001), but negatively correlated with negative affect (r = − 0.23, − 0.31 and − 0.38, *ps* < 0.001), depression (r = − 0.22, − 0.30 and − 0.32, *ps* < 0.001), compulsion-anxiety (r = − 0.22, − 0.34 and − 0.40, *ps* < 0.001) and perceived stress (r = − 0.38, − 0.43 and − 0.43, *ps* < 0.001).

**Table 4:** Correlations between PCOISS and criterions (n_A_ = 1355)

	C-PCOISS	Factor 1	Factor 2	Factor 3
Positive affect	0.35***	0.37***	0.33***	0.16***
Negative affect	− 0.36***	− 0.23***	− 0.31***	− 0.38***
Depression	− 0.34***	− 0.22***	− 0.30***	− 0.32***
Compulsion-anxiety	− 0.38***	− 0.22***	− 0.34***	− 0.40***
Perceived stress	− 0.49***	− 0.38***	− 0.43***	− 0.43***

Significance is indicated by (^***^) for *p* < 0.001

### Reliability analysis

Table 5 showed the internal consistency and test–retest reliability of this 14-item PCOISS. The Cronbach’s alpha coefficients were individually 0.86, 0.78 and 0.72 for three subscales of the C-PCOISS (i.e., ‘having the techniques for control of internal states’, ‘sense of efficacy of controlling internal states’ and ‘sense of lack of efficacies’), respectively. We collected 761 retest responses from Data_B_. The C-PCOISS also demonstrated high internal consistency (all Cronbach’s alphas > 0.75) and correlation between test and retest on three subscale scores (r = 0.64, 0.62 and 0.54, *ps* < 0.001).

**Table 5:** The reliabilities in each factor and the C-PCOISS

	14-item PCOISS	Factor 1	Factor 2	Factor 3
internal consistency reliability (n_A_ = 1355)	0.91	0.86	0.78	0.72
test–retest correlation (n_C_ = 761)	0.70***	0.64***	0.62***	0.54***

Significance is indicated by (^***^) for *p* < 0.001

## Discussion

This is the first study to translate the PCOISS into Chinese and validate its psychometric properties in a large sample of Chinese adults. The findings showed that the C-PCOISS had good validity and reliability. PCA and CFA results jointly supported an independent three-factor structure for the 14-item C-PCOISS. The scale had good convergent validity with reasonable standardized factor loadings (> 0.50) and composite reliabilities (> 0.70) [51], and satisfactory discriminant validity revealed by the best fit values in model comparisons [41]. In terms of criterion validity, the C-PCOISS showed positive correlations with positive affect and negative correlations with negative affect, depression, compulsion-anxiety and perceived stress, which was consistent with prior literature [19, 27, 52, 53]. Last but not least, adequate internal consistency (Cronbach’s alpha coefficient = 0.91) and test-retest reliability (Pearson correlation coefficient = 0.70) ensured that the scale had high homogeneity and one-month temporal stability in China. Taken together, these findings suggested that the C-PCOISS is valid for assessing perceived control of internal states.

The C-PCOISS demonstrated comparable three-factor construct to the original English version [27] and the adapted Turkish version [28]. Specifically, the cumulative variance contribution rate (58.93%) was comparable to 58.50% achieved in the original version [27], and higher than 47.80% in the Turkish version [28]. Both the Chinese and the Turkish versions [28] found that the three-factor model (i.e., ‘having the techniques for control of internal states’, ‘sense of efficacy of controlling internal states’ and ‘sense of lack of efficacies’) was supported with the best fit (i.e., GFI, CFI and RMSEA) among several alternative models. As the English version didn’t conducted CFA [27], we failed to make comparison of CFA results between the three versions. For the internal consistency, the Cronbach’s alpha coefficient in the Chinese version (0.91) was similar to that in the original English version (0.92) [27] and higher than that in the Turkish version (0.85) [28].

There are some differences for several specific items among the original English version (18 items) [27], the Turkish version (16 items) [28] and the C-PCOISS (14 items). Comparing to the English version with 18 items, item 1, 2, 6 and 8 were removed in the Chinese version due to poor cross-loadings values (> 0.40), while in the Turkish version [28], item 9 was removed since it was available in all three factors, and item 16 was excluded as it measured concepts similar to that of item 15 but had a lower fit value. The cross-cultural difference is one possible reason for this discrepancy. According to the Cultural Model of Emotions [54], different models of emotional regulation depend on specific social contexts. For example, the interpersonal harmony is highly advocated in Chinese culture [55], which may cause that some Chinese adults answered negative-wording items about emotion regulation (e.g., item 1 and item 2) with potential bias to preserve social harmony [56, 57]. In addition, it may be difficult to distinguish item 6 and 8 between different dimensions in Chinese context. For example, the description “distract myself and think about something nicer” in item 8 not only emphasizes that individuals possess techniques for controlling thoughts, but also is related to sense of efficacy when putting it into the context “start to worry about something”. Age-group differences may be the other reason. Adults at least 20 years old were recruited in the Chinese and the English versions [27], while adolescents with a mean age of 15.71 years were surveyed in the Turkish version [28]. As the C-PCOISS was determined in a relatively representative sample size which was four times as large as the English version [27] and six times larger than the Turkish version [28], it was convincing that the 14-item C-PCOISS was robustly verified and applicable to Chinese adults.

The C-PCOISS has great potential both in theory and practical application. Theoretically, we adapted the measurement of perceived control over internal states into China for the first time, aiming to enrich the connotation of internal control besides the locus of control theory [58]. An interesting research theme is to explore what role the perceived control over internal states plays in boosting resilience based on the tripartite model of resilience-building [59]. On the application, the PCOISS has been found to help identify individuals vulnerable to mental health problems [13–16] and evaluate the effectiveness of some clinical interventions or treatments [22, 23]. Therefore, the C-PCOISS will help fill in gaps in some fields like stress management, psychological counseling, public health and mental disorders in China.

Several limitations should be noted. Firstly, although the C-PCOISS was verified in a large sample across a wide-age, it employed a convenience sampling method of the online survey that was not feasible for accessing individuals without smartphones or computers, so the present results may cause bias when generalized to the whole population. Secondly, the C-PCOISS was tested only among Chinese adults in the context of COVID-19 pandemic. In the future, researchers can consider using stratified random sampling to examine psychometric properties in diverse groups (e.g., children, adolescents, the elderly and clinical samples) and in non-specific usual time. Thirdly, the criteria of interest used in the present study were all self-reported questionnaires, more ecological criteria such as behavior performances and physiological signals could be involved in further work. Finally, internal states in the present scale mainly focused on emotion, thoughts and physical reactions, more components of internal states (e.g., motivation, and arousal) may be integrated to improve the structure in further studies.

## Conclusions

The PCOISS is adapted in the Chinese context. Our findings showed that the C-PCOISS had adequate validity and reliability among Chinese adults, indicating that the scale is a feasible instrument to measure perceived control of internal states in China. Future research is needed to verify the applicability of the C-PCOISS in different fields such as mental health and stress management in China.

## Supplementary Information

**Additional file 1.** Details of the final version of the C-PCOISS and results including item analysis and random intercept factor model.

## Data Availability

The datasets used and/or analysed during the current study available from the corresponding author on reasonable request.

## Abbreviations

AIC: Akaike’s information criterion
BIC: Bayesian information criterion
CAIC: Consistent AIC
CFA: Confirmatory factor analyses
CFI: Comparative fit index
C-PCOISS: The Chinese version of the Perceived Control of Internal States Scale
GFI: Goodness-of-fit index
GSES: The Generalized Self-Efficacy Scale
KMO: Kaiser–Meyer–Olkin
I-E Scale: The Internal–External Locus of Control Scale
PANAS: The Positive and Negative Affect Schedule
PCA: Principal component analysis
PCOISS: The Perceived Control of Internal States Scale
PQEEPH: The Psychological Questionnaire for Emergent Events of Public Health
PSS10: The Perceived Stress Scale 10-item version
RMSEA: Root mean square error of approximation
SARS-PBQ: The SARS Psychological Behavior Questionnaire
SRMR: Standardized root mean square residual
TLI: Tucker-Lewis index
