# Supplementary material for: Adaption and validation of the Perceived Control of Internal States Scale (PCOISS) in Chinese adults: a cross-sectional study
Source: BMC Psychol. 2022 Dec 5;10:290. doi: 10.1186/s40359-022-01004-2 (PMC9724291; doi:10.1186/s40359-022-01004-2)
Supplement: Supplementary file 1 — Additional file 1. Details of the final version of the C-PCOISS and results including item analysis and random intercept factor model. [file 40359_2022_1004_MOESM1_ESM.docx]

Supplementary materials

Appendix 1 The Chinese version of the PCOISS (C-PCOISS)

Supplemental Table 1 The initial 18 items of the C-PCOISS

| Perceived Control of Internal States Scale (adapted from Pallant [[27](#_ENREF_27)])  Instructions: Read the scale provided and decide how much you either disagree or agree with each of the following statements according to your recent actual situation. Circle the number from 1 to 5 that best indicates how you feel: 1 = strongly disagree, 2 = disagree, 3 = neutral, 4 = agree, 5 = strongly agree. | | | | | |
| --- | --- | --- | --- | --- | --- |
| *1. I don’t have much control over my recent emotional reactions. | 1 | 2 | 3 | 4 | 5 |
| *2. When I have been in a bad mood recently I find it hard to snap myself out of it. | 1 | 2 | 3 | 4 | 5 |
| 3. My feelings have usually been fairly stable recently. | 1 | 2 | 3 | 4 | 5 |
| 4. I can talk myself out of feeling bad. | 1 | 2 | 3 | 4 | 5 |
| 5. No matter what happens to me in my life I am confident of my ability to cope emotionally. | 1 | 2 | 3 | 4 | 5 |
| 6. I have a number of good techniques that will help me cope with any stressful situation. | 1 | 2 | 3 | 4 | 5 |
| *7. I find it hard to stop myself from thinking about my problems. | 1 | 2 | 3 | 4 | 5 |
| 8. If I start to worry about something I can distract myself and think about something nicer. | 1 | 2 | 3 | 4 | 5 |
| 9. If I realise I am thinking silly thoughts I can stop myself. | 1 | 2 | 3 | 4 | 5 |
| 10. I am able to keep my thoughts under control. | 1 | 2 | 3 | 4 | 5 |
| *11. I imagine there will be many situations in the future where silly thoughts will get the better of me. | 1 | 2 | 3 | 4 | 5 |
| 12. I have a number of techniques which I am confident will help me think clearly and rationally in any situation I might find myself. | 1 | 2 | 3 | 4 | 5 |
| 13. Even when under pressure I can usually keep calm and relaxed. | 1 | 2 | 3 | 4 | 5 |
| 14. I have a number of techniques or tricks that I use to stay relaxed in stressful situations. | 1 | 2 | 3 | 4 | 5 |
| *15. When I’m anxious or uptight there does not seem to be much that I can do to help myself relax. | 1 | 2 | 3 | 4 | 5 |
| *16. There is not much I can do to relax when I get uptight. | 1 | 2 | 3 | 4 | 5 |
| 17. I have a number of ways of relaxing that I am confident will help me cope. | 1 | 2 | 3 | 4 | 5 |
| 18. If my stress levels get too high I know there are things I can do to help myself. | 1 | 2 | 3 | 4 | 5 |

Note. Bold represents the final 14 items comprising the C-PCOISS; * indicates reverse coded items.

Key:

having the techniques for control of internal states = 12, 13, 14, 17, 18;

sense of efficacy of controlling internal states = 3, 4, 5, 9, 10;

sense of lack of efficacies = 7, 11, 15, 16.

Appendix 2 Item analysis

The mean, standard deviation, critical ratio (CR) and corrected item-total correlation of each item are shown in the [Supplemental Table 2](#S2).

Supplemental Table 2 Descriptive information and discrimination test’s results of each item (n_A_=1355)

| Item number | Mean | SD | Critical ratio (CR) | Corrected  item-total correlation |
| --- | --- | --- | --- | --- |
| 1 | 3.75 | 0.93 | 22.63^***^ | 0.52 |
| 2 | 3.79 | 1.1 | 24.95^***^ | 0.58 |
| 3 | 3.76 | 0.91 | 20.67^***^ | 0.52 |
| 4 | 3.90 | 0.80 | 23.11^***^ | 0.60 |
| 5 | 3.94 | 0.82 | 24.78^***^ | 0.64 |
| 6 | 3.59 | 0.90 | 26.72^***^ | 0.61 |
| 7 | 3.24 | 1.03 | 16.13^***^ | 0.36 |
| 8 | 3.65 | 0.84 | 19.45^***^ | 0.52 |
| 9 | 3.74 | 0.84 | 20.20^***^ | 0.53 |
| 10 | 3.78 | 0.81 | 25.21^***^ | 0.61 |
| 11 | 3.69 | 1.02 | 16.97^***^ | 0.40 |
| 12 | 3.53 | 0.88 | 25.84^***^ | 0.60 |
| 13 | 3.59 | 0.89 | 28.87^***^ | 0.65 |
| 14 | 3.43 | 0.93 | 27.54^***^ | 0.64 |
| 15 | 3.60 | 0.98 | 24.95^***^ | 0.58 |
| 16 | 3.58 | 1.02 | 26.49^***^ | 0.58 |
| 17 | 3.48 | 0.88 | 27.30^***^ | 0.63 |
| 18 | 3.76 | 0.81 | 24.04^***^ | 0.60 |

Note. Significance is indicated by (^***^) for *p* < 0.001.

Appendix 3 Random intercept factor model

In random intercept factor model (Model 3, see [Supplemental Table 3](#S3)), trait factor loading was significant (*p* < 0.001) and greater than method factor loading in each item. Model 3 had an acceptable fit to the data with the rational ratio between trait variance and the method effect variance (1 : 0.076). Method effect variance was larger than its standard error (Estimate = 0.076, S.E. = 0.005, z = 16.64, *p* < 0.001). These results indicate the 14-item PCOISS is not affected by the wording effect.

Supplemental Table 3 Factor loadings of random intercept factor model (n_B_ = 1354)

| Item | Trait factor | Method factor |
| --- | --- | --- |
|  | Estimate (S.E.) | Estimate |
| 12 | 0.6 (0.02)^***^ | 0.33 |
| 13 | 0.67 (0.02)^***^ | 0.30 |
| 14 | 0.65 (0.02)^***^ | 0.29 |
| 17 | 0.65 (0.02)^***^ | 0.31 |
| 18 | 0.59 (0.02)^***^ | 0.33 |
| 3 | 0.45 (0.03)^***^ | 0.29 |
| 4 | 0.56 (0.02)^***^ | 0.33 |
| 5 | 0.57 (0.02)^***^ | 0.34 |
| 9 | 0.46 (0.03)^***^ | 0.31 |
| 10 | 0.59 (0.02)^***^ | 0.31 |
| 7 | 0.46 (0.03)^***^ | –0.25 |
| 11 | 0.57 (0.03)^***^ | –0.26 |
| 15 | 0.73 (0.03)^***^ | –0.27 |
| 16 | 0.69 (0.03)^***^ | –0.26 |

Note. Significance is indicated by (^***^) for *p* < 0.001.
